# Transplantation of Autologous Adipose Stem Cells Lacks Therapeutic Efficacy in the Experimental Autoimmune Encephalomyelitis Model

**DOI:** 10.1371/journal.pone.0085007

**Published:** 2014-01-21

**Authors:** Xiujuan Zhang, Annie C. Bowles, Julie A. Semon, Brittni A. Scruggs, Shijia Zhang, Amy L. Strong, Jeffrey M. Gimble, Bruce A. Bunnell

**Affiliations:** 1 School of Petroleum & Chemical Engineering, Dalian University of Technology Panjin Campus, Panjin, Liaoning Province, China; 2 Center for Stem Cell Research and Regenerative Medicine, Tulane University School of Medicine, New Orleans, Louisiana, United States of America; 3 Department of Cell and Molecular Biology, Tulane University School of Science and Engineering, New Orleans, Louisiana, United States of America; 4 Department of Pharmacology, Tulane University School of Medicine, New Orleans, Louisiana, United States of America; 5 Stem Cell Biology Laboratory, Pennington Biomedical Research Center, Baton Rouge, Louisiana, United States of America; 6 Division of Regenerative Medicine, Tulane National Primate Research Center, Covington, Louisiana, United States of America; Friedrich-Alexander University Erlangen, Germany

## Abstract

Multiple sclerosis (MS), characterized by chronic inflammation, demyelination, and axonal damage, is a complicated neurological disease of the human central nervous system. Recent interest in adipose stromal/stem cell (ASCs) for the treatment of CNS diseases has promoted further investigation in order to identify the most suitable ASCs. To investigate whether MS affects the biologic properties of ASCs and whether autologous ASCs from MS-affected sources could serve as an effective source for stem cell therapy, cells were isolated from subcutaneous inguinal fat pads of mice with established experimental autoimmune encephalomyelitis (EAE), a murine model of MS. ASCs from EAE mice and their syngeneic wild-type mice were cultured, expanded, and characterized for their cell morphology, surface antigen expression, osteogenic and adipogenic differentiation, colony forming units, and inflammatory cytokine and chemokine levels *in vitro*. Furthermore, the therapeutic efficacy of the cells was assessed *in vivo* by transplantation into EAE mice. The results indicated that the ASCs from EAE mice displayed a normal phenotype, typical MSC surface antigen expression, and *in vitro* osteogenic and adipogenic differentiation capacity, while their osteogenic differentiation capacity was reduced in comparison with their unafflicted control mice. The ASCs from EAE mice also demonstrated increased expression of pro-inflammatory cytokines and chemokines, specifically an elevation in the expression of monocyte chemoattractant protein-1 and keratin chemoattractant. *In vivo*, infusion of wild type ASCs significantly ameliorate the disease course, autoimmune mediated demyelination and cell infiltration through the regulation of the inflammatory responses, however, mice treated with autologous ASCs showed no therapeutic improvement on the disease progression.

## Introduction

Multiple sclerosis (MS), characterized by chronic inflammation, demyelination, and axonal damage, is a complicated neurological disease of human central nervous system (CNS) and is the most common neurological disease in young adults among 20–40 years old [1]–[3]. So far, immunotherapies are the most commonly used treatment approaches for MS, ranging from nonselective immunosuppressor, such as interferon-β or glatiramer acetate, to highly specific immune interventions such as the monoclonal antibody natalizumab against the cell adhesion molecule α4-integrin [2], [3]. Although the existing treatments for MS partially alleviate symptoms, they do not halt the ongoing progression of neurodegeneration. Experimental autoimmune encephalomyelitis (EAE), which exhibits many clinical and histological features of MS, is the most widely used and best characterized experimental animal model for MS. EAE is usually induced by immunization with myelin proteins such as myelin oligodendrocyte glycoprotein (MOG) and adjuvants, which results in the immune system mediated destruction of the myelinated CNS [4], [5].

Bone marrow-derived mesenchymal stem cell (BM-MSC) based cell therapy for EAE have been extensively studied [6]–[12] because of the BM-MSCs' self-renewal ability, potential of differentiation into multiple lineages, and immunomodulatory effects through the secretion of cytokines and chemokines. Although bone marrow remains the main source for mesenchymal stem cells in clinical trials, adipose tissue is an ideal source due to its abundance, easy accessibility, and the feasibility of harvesting by a minimally invasive procedure. Moreover, large amounts of cells can be isolated from the adipose tissue, yielding 100–500 fold higher stem cells per tissue volume than bone marrow [13], [14]. Meanwhile, adipose-derived stromal/stem cells (ASCs) have similar self-renewal abilities, surface epitopes, growth kinetics, and cytokine expression profiles as their counterparts from bone marrow [15]–[20].

Studies have been performed using ASC or genetically-modified ASC therapy for the treatment of EAE [14], [21]. The results from these studies clearly demonstrated that ASC-based cell therapy could ameliorate the disease and enhance anti-inflammatory responses. Consequently, it is important to consider the question of whether the outcome of ASCs infusion differs when cells are sourced from EAE afflicted or unaffected controls. It is presumed that autologous ASC transplantation will have lower rates of post-transplant complications and lower risk of transmission of infectious diseases compared to allogeneic transplantation. Moreover, although allogeneic MSC therapy has been used for the treatment of various diseases, such as Graft versus host disease, some studies have shown that allogeneic MSCs transplantation could be rejected by the host immune system [22]. In contrast, autologous transplantation of stem cells does not have issues related to donor availability or risk of histo-incompatibility. Thus, autologous ASCs offer numerous theoretical advantages from regulatory, histocompatibility, and immunological perspectives [23].

In the *in vitro* studies presented here, ASCs were isolated from diseased EAE mice, cultured, expanded, and characterized by means of flow cytometry, differentiation assays, colony forming unit assays, and real-time PCR to determine whether the EAE alters ASCs properties relative to those derived from unafflicted control mice. *In vivo*, ASCs from the EAE mice, transplanted back in the EAE mouse model, were assessed for their therapeutic effects on delaying the disease course, ameliorating the demyelination and modulation of the immune responses.

## Materials and Methods

### EAE induction and treatment

All the protocols and experimental procedures were approved by the Institutional Animal Care and Use Committee at Tulane University. Female C57Bl/6 mice, 6 to 8 weeks old, were purchased from Charles River Laboratories (Wilmington, MA), and allowed 7 days to acclimate before the start of the study. Chronic EAE was induced in the female C57Bl/6 mice by subcutaneous immunization with MOG_35–55_ (Anaspec, San Diego, CA), which was emulsified in Complete Freund's adjuvant with 8 mg/ml of Mycobacterium tuberculosis H35RA (Difco, Detroit, MI) in 1∶1 ratio, and 100 µl (100 ng) of emulsified MOG_35–55_ was injected sub-cutaneously at each side of the base of the tail respectively. Each mouse also received 100 µl (200 ng) of pertussis toxin (List Biological Laboratories, Campbell, CA) by intraperitoneal (i.p.) injection, and the injection of toxin was repeated 2 days later. Concurrently, a total of 1×10^6^ ASCs from the EAE mice (EAEASC) or from unafflicted control mice (WtASC) suspended in 100 µl total volume of HBSS were injected into the mice intraperitoneally. Negative control animals consisted of EAE-induced mice injected with equal volumes of vehicles only (HBSS), and positive control animals were non-EAE-induced normal mice which only received one i.p. injection of the equal volume of HBSS. The experiments were conducted on three independent cohorts with 20 mice total for each treatment and control group.

### Isolation and culture of adipose-derived stem cells

The subcutaneous inguinal fat pads were isolated from the Hank's balanced salt solution (HBSS) (Life Technologies, Grand Island, NY)-treated EAE mice and HBSS treated normal non-induced mice on post EAE disease induction (PDI) day 30, rinsed with HBSS to remove blood and hair contamination, and digested with 0.1% collagenase type 1 solution (Life Technologies) for approximately 4 hours at 37°C under mild agitation. The digested adipose tissues were then filtered through a 70-µm nylon mesh cell strainer (BD Biosciences, Bedford, MA), and centrifuged at 500×*g* for 10 minutes at room temperature (RT). The pellets were re-suspended and cultured in the cell culture medium (CCM), made of DMEM∶F12 (Life Technologies) supplemented with 10% fetal bovine serum(FBS, Atlanta Biologicals, Atlanta, GA), 2 mM L-glutamine (Life Technologies), and 1% antibiotic/antimycotic (Penicillin/streptomycin/amphotericin, Life Technologies). After 24 hours, the non-adherent cells were washed with phosphate buffered saline (PBS, Life Technologies) and fresh CCM were added. When cells reached 70–80% confluence, the passage 0 cells were lifted with 0.25% trypsin/1 mM EDTA (Life Technologies) and sub-cultured at 100 cells/cm^2^ in CCM on 145 cm^2^ tissue culture dishes (Nalge Nunc International, Rochester, NY). Media was replaced every 3–4 days, and cells were routinely passaged when they reached 70% confluence unless otherwise noted. For all experiments, ASC lines between passages 3–5 were used.

### Flow cytometry

The following antibodies were used to check the cell surface marker profiles of EAEASCs and WtASCs: CD29, CD34, CD31, CD45, CD11b, and Sca1 (stem cell antigen-1). All of the antibodies were purchased from BD Biosciences. The ASCs were cultured, trypsinized, pelleted, and re-suspended in 500 µl PBS. The cells were incubated with the antibodies for 30 minutes at RT, then washed with PBS, and analyzed by Cytomics FC500 (Beckman Coulter, Brea, CA). The results were analyzed with CXP analysis software (Beckman Coulter).

### Differentiation

ASCs at passage 5 were cultured on 6-well Nunc plates (Nalge Nunc International) to approximately 90% confluence before adipogenic and osteogenic differentiation media were added. Adipogenic differentiation medium was made with CCM supplemented with 5 µg/ml insulin, 50 µM indomethacin, 1 µM dexamethasone and 0.5 µM 3-isobutyl-1-methylxanthine (all media supplements were purchased from Sigma, St Louis, MO). Osteogenic differentiation medium was made with CCM supplemented with 1 nM dexamethasone, 20 mM β-glycerolphosphate, 50 µM L-ascorbic acid 2-phosphate sesquimagnesium salt, and 50 ng/ml L-thyroxine sodium pentahydrate. Media were changed twice per week for 3 weeks. For adipogenic differentiation, the cells were washed with PBS, fixed with 10% formalin (Sigma) for 20 minutes at RT, washed again with PBS, stained with Oil Red-O (Sigma) for 20 minutes at RT, and washed with PBS until wash was clear. For the detection of osteogenesis, the cells were washed with PBS, fixed with 10% formalin for 20 minutes at RT, washed with deionized (DI) water, stained with Alizarin Red (Sigma) for 20 minutes at RT, and washed with DI water until wash was clear. Images were acquired at 10× for adipogenic differentiation and 4× for osteogenic differentiation on Nikon Eclipse TE200 (Melville, NY) with Nikon Digital Camera DXM1200F using the Nikon ACT-1 software version 2.7.

The levels of adipogenic and osteogenic differentiation were also quantified. For the quantification of adipogenic differentiation, the accumulated lipids were eluted with isopropanol after images were captured. The amount of Oil Red O was measured by recording the optical density (OD) of the solution at 584 nm. The results were normalized to the protein content of the samples with the BCA assay (Thermo Scientific, Rockford, IL). For the quantitative osteogenesis assay, the cells were de-stained, after images were taken, with 10% cetylpyridinium chloride (Sigma) for 30 minutes at RT. The amount of Alizarin Red was determined by measuring the OD of the solution at 584 nm. The results were normalized to the protein content of the samples.

### Colony forming unit assay

ASCs at passage 5 were seeded onto 56.7 cm^2^ Nunc cell culture plates (Nalge Nunc International) in 5 replicates at a total of 100 cells per plate. Growth media was changed every 3–4 days. After 14 days, the cells were washed with PBS, stained with 3% crystal violet in 100% methanol for 30 minutes at RT, and then washed with DI water at least 3 times to remove excess dye. All colonies greater than 2 mm in diameter were counted. The cells were de-stained with methanol, after images were taken, for 40 minutes under mild agitation at RT. The amount of staining was measured by recording the optical density (OD) of the solution at 584 nm, and the arbitrary unit was defined as the OD divided by the pre-counted colony number.

### Real-time quantitative PCR

Cells at passage 5 were seeded onto 145 cm^2^ tissue culture dishes with a density of 1×10^6^ cells/dish, and incubated for 24 hours. The cells were then trypsinized and pelleted for extraction total cellular RNA using RNeasy Mini Kit (Qiagen, Valencia, CA). The concentration and purity of the RNA were assessed by Nanodrop 2000 spectrophotometer (Thermo Scientific). RNA was first treated with DNase (Life Technologies), and first strand cDNA syntheses were performed using iScript cDNA Synthesis kit (Biorad, Hercules, CA). Real-time PCR was performed to analyze the mRNA levels for mouse cytokines and chemokines, specifically TNFα, IL-6, MCP-1, MIP-1α, RANTES, KC, MIP-2α and VEGF. PCR reactions were performed using CFX96 Real Time System (Biorad) in a total volume of 20 µl containing 10 µl Taqman mastermix (Applied Biosystems, Foster City, CA), 1 µl primer and probe mix (Applied Biosystems), 7 µl dH_2_O and 2 µl template. Reaction mixtures were incubated at 50°C for 2 minutes and 95°C for 10 minutes, and reactions were allowed to proceed via 40 cycles of melting at 95°C for 15 seconds, annealing and extension at 60°C for 1 minute. The housekeeping gene β-actin (UniGene: Mm328431) was used as internal reference. Quantification was calculated using ΔΔCt method [24].

### Clinical Scoring

Mice were monitored daily for clinical signs of EAE by three independent investigators. Clinical scores were based on a scale of 0–5 with a score of 0 indicating no disease; 1-limp tail (loss of tail tone); 2-limp tail and hind limb weakness; 3-limp tail and partial hind limb paralysis; 4-limp tail and complete hind limb paralysis; and 5- moribund or dead.

### Tissue Processing and Histological Analysis

On PDI day 30, animals were euthanized by exposure to CO_2_ and perfused with sterile PBS. Spinal cords were removed, fixed in 10% formalin (Thermo Scientific) and then embedded in paraffin. Sections were cut at 5 microns on a microtome and stained for Luxol Fast Blue/Crystal Violet (LFB, IHC World, Ellicott City, MD) and Hematoxylin and Eosin (H&E) for identification of intact myelin and infiltrating cells respectively. Histological images were collected on an ImageScope (Aperio, Vista, CA), and the LFB images were analyzed with Aperio Software. The quantification of the percentage of positivity was determined by the percentage of positive pixels divided by the total number of pixels in a given section. The H&E images were analyzed with Fiji/Image J software to quantify the number of total cells (i.e., cells ≥5 µm^2^) per field at 40× magnification [25]. Quantification was performed on three sections per animal and three animals per group. Indices were normalized to the average value obtained in normal un-induced mice (set as 1).

### Analysis of Spinal Cord Lesions

Spinal cord sections were prepared 5 microns thick and stained with LFB and H&E for myelination and infiltrating cell detection, respectively. Each section was analyzed using Aperio ImageScope for quantitative measures of lesions. Lesion detection was determined by dense areas of both LFB and H&E stain containing high concentrations of infiltrating cells located in the ventral and lateral columns of the spinal cord. Lesions were manually delineated in H&E stained sections and subsequently analyzed using modified positive pixel count algorithms specific for H&E stained sections.

Lesions were scored according to categories established in Niimi et al., 2013 [26]. Briefly, Grade 1- leptomeningeal cell infiltration, Grade 2- mild perivascular cuffing, Grade 3- moderate perivascular cuffing, Grade 4 – extensive perivascular cuffing.

### Immunohistochemistry

Tissue sections (paraffin-embedded) were warmed on a heating platform at 57°C for 30 minutes prior to deparaffinization. For deparaffinization, all slides were submerged in HistoChoice (Amresco, Solon, OH) twice for 5 min, 100% ethanol twice for 2 min, 95% ethanol twice for 2 min, 70% ethanol for 2 min, 50% ethanol for 2 min, and DI water twice for 2 min. The deparaffinized slides were submerged in citrate buffer pH 6.0 (10 mM) and heated for 30 min in a steamer. After cooling, the slides were washed for 5 min in 1× PBS and subsequently washed with PBS-FSG-Tx-100 (10% v/v 10× PBS, 0.2% v/v fish skin gelatin, and 0.1% v/v Triton x-100) for 5 min before incubation for 1 h in a humidified chamber at RT with blocking solution, which consisted of 10% normal goat serum (NGS) in PBS-FSG (10% v/v 10× PBS and 0.2% v/v fish skin gelatin). The primary antibody to astrocytes (GFAP; 1∶200, Sigma: C9205 Ms IgG1), CD3 (1∶200, Abcam, Cambridge, MA), CD11b (1∶200, eBiosciences, San Diego, CA), CD45 (1∶200, Invitrogen, Grand Island, NY) was diluted in 10% NGS solution and applied to appropriate experimental sections for overnight incubation in a humidified chamber at 4°C. Control slides were treated with secondary antibody-only (2° only). Following incubation, the slides were washed in PBS-FSG-Tx-100 and PBS-FSG, each for 10 min. The sections stained with anti-GFAP were then incubated in a humidified chamber at RT for 1 hour with the secondary antibody (e.g., anti-rat, with Alexa 594) in 10% NGS solution. Slides were then washed twice in PBS-FSG-Tx-100 and once in PBS-FSG. The slides were mounted with mounting medium with DAPI (Vector Laboratories, INC. Burlingame, CA), and imaged using a deconvolution microscope. Slides, stained with anti-CD3, CD11b, and CD45, were incubated with HRP-conjugated secondary antibodies (Abcam, Cambridge, MA) for 1 hour at room temperature then processed with DAB peroxidase substrate kit (Vector Laboratories, Inc., Burlingame, CA) followed by Hematoxylin (Invitrogen, Grand Island, NY) counterstain. Slides were washed, dehydrated, and mounted with Permount (FisherChemicals, Fair Lawn, NJ).

Spinal cord sections analyzed with ImageScope were used to identify CD3, CD11b, and CD45-positive cells within the manually delineated lesions for each group. Using the positive pixel count algorithm, the frequency of each antigen-specific cell infiltrating within each lesion was determined by the number of strong positive pixels (Nsp) divided by the total number of pixels (NTotal) multiplied by 100.

### Serum ELISA assay

On PDI day 30, blood samples were collected from each treatment and control group to analyze circulating levels of cytokines TNFα, IL-12 and IL-17 by ELISA assay (Invitrogen, Grand Island, NY) according to the manufacturer's instructions. The concentration of each cytokine was calculated based on the standard curve generated.

### Statistical analysis

Data sets were analyzed by the Kruskal-Wallis non-parametric one-way analysis of variance (ANOVA), followed by post-hoc Dunnet multiple comparison tests versus the respective control group. For pair-wise comparisons, the F-test was used to determine whether a given pair of population variances was equal (α<0.05). This information was then used in designating the appropriate t-tests (typically heteroscedastic) to perform for comparing the means of population pair, with significance defined as *P*<0.05. All values were reported as mean ± SD except for the clinical scoring where the values were reported as means ± SEM.

## Results

### Flow cytometric analysis of EAEASC

**Figure 1:**
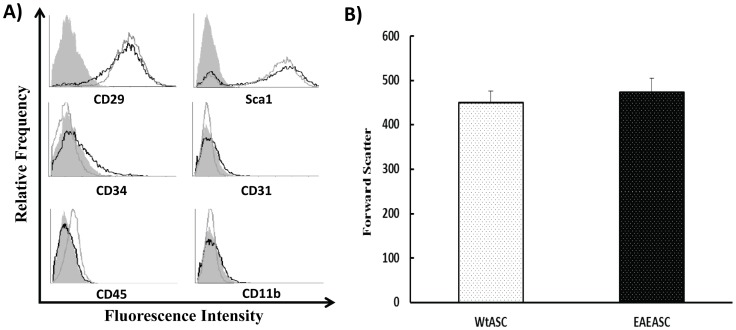
Cell surface marker profiles of EAEASCs and WtASCs. A) Cells were analyzed by flow cytometry for MSC surface markers CD29, Sca1; hematopoietic markers CD34 and CD45; phagocytic lineage marker CD11b; and endothelial marker CD31. Gray filled: isotype control; gray line: WtASCs; black line: EAEASCs. B) Cell size based on forward scatter signal of flow cytometry (*P* = 0.26, t-test, n = 7).

The cell morphology of EAEASCs was consistent with that of the ASCs from the unafflicted control mice, which were fibroblast-like in appearance (data not shown). EAEASCs and WtASCs were analyzed by flow cytometry, and their cell surface antigens were similar (Fig. 1A). Both cell types were positive for CD29 and ScaI, while they were negative for endothelial (CD31), hematopoietic markers (CD45 and CD34) and macrophage marker CD11b. The sizes of both cell types were also analyzed based on the forward scatter signals of flow cytometry. As shown in Figure 1B, the cell size of EAEASCs versus WtASCs was not significantly different (t-test, *P*>0.05).

### Differentiation assays for EAEASC

**Figure 2:**
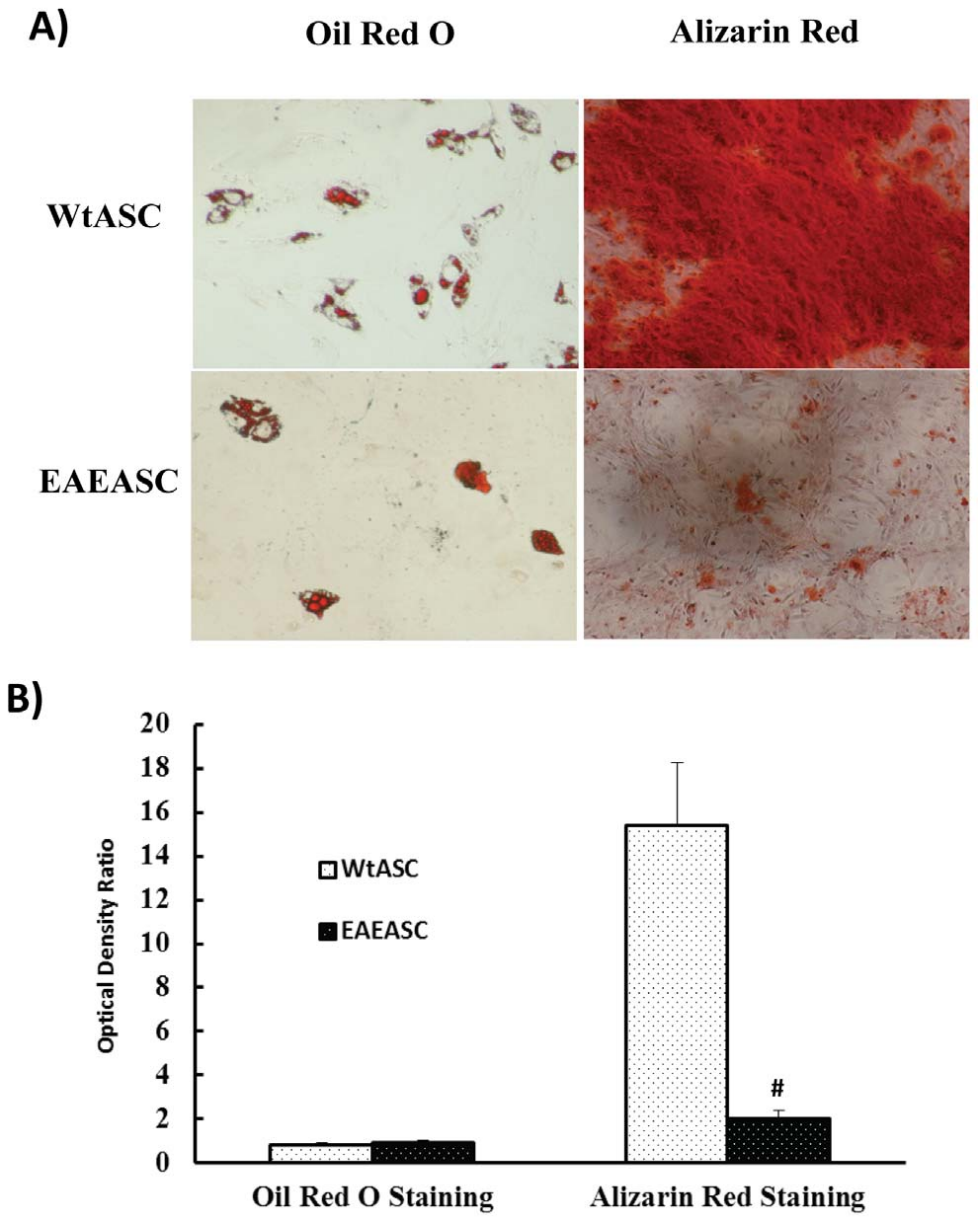
Analysis of differentiation of EAEASCs and WtASCs along osteogenic and adipogenic lineages. A) Differentiated EAEASCs and WtASCs. Cells were incubated in adipogenic or osteogenic differentiation media for 21days and stained, respectively, with Oil Red O and Alizarin Red. For osteogenic differentiation, images were collected at 4× magnification. For adipogenic differentiation, images were obtained at 10× magnification. B) Quantification of osteogenesis and adipogenesis. For the quantitation of osteogenesis, the cells were de-stained with 10% cetylpyridinium chloride after stained with Alizarin Red. For the quantitation of adipogenesis, the cells were de-stained with isopropanol. Optical density (OD) was measured at 584 nm and normalized to protein content. The bar graph represents the ratio of normalized OD of differentiated cells and normalized OD of control cells. # means *P*<0.05 vs WtASCs (t-test, n = 3).

EAEASCs and WtASCs were cultured in osteogenic and adipogenic differentiation medium for 3 weeks to test their lineage differentiation efficiency. On day 21, the ASCs were stained with Alizarin Red to assess bone mineralization, and with fresh Oil Red O for lipid droplets. Both EAEASCs and WtASCs efficiently differentiated into osteocytes and adipocytes (Fig. 2). However, as shown in Figure 2A, the EAEASCs had reduced osteogenic differentiation capacity when compared to the WtASCs. Quantification of the differentiation levels of both cell types (Fig. 2B) further demonstrated that WtASCs (OD ratio = 15.39±2.88) could differentiate into osteocytes to a much greater degree than EAEASCs (OD ratio = 2.03±0.35) (t-test, *P*<0.05) over the period of the assay. Quantification of adipocyte differentiation indicated that EAEASCs (OD ratio = 0.90±0.11) had a similar differentiation capacity as WtASCs (OD ratio = 0.80±0.14) (t-test, *P*>0.05). The graph in Figure 2B represents the ratios of OD of differentiated cells normalized to control cells. Real-time PCR analysis of osteogenic markers alkaline phosphatase and runt-related transcription factor 2 in differentiated EAEASCs and WtASCs showed that there was no difference in these two transcriptional factors between the two cell types (data not shown).

### Colony forming unit assay

**Figure 3:**
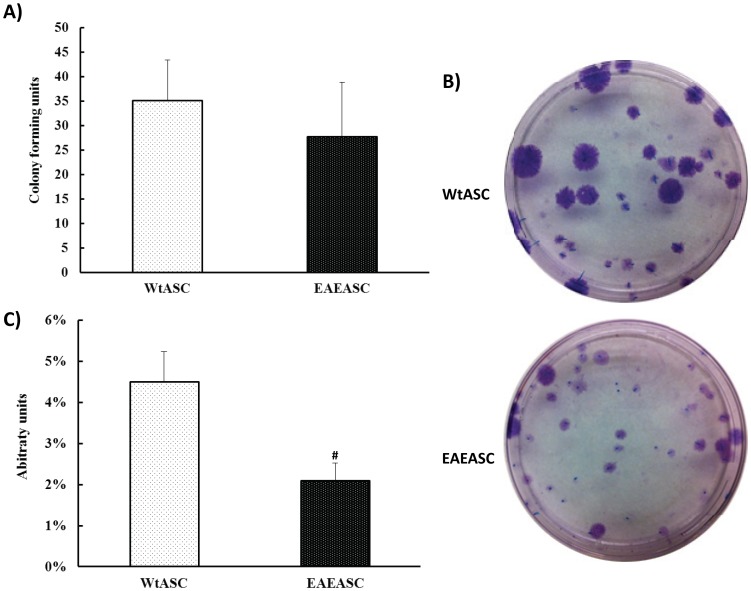
Colony forming unit assays for EAEASCs and WtASCs. A total of 100 cells were plated on 56.7^2^ Nunc cell culture plates and incubated for 14 days. Cells were stained with 3% crystal violet, and colonies 2 mm or larger in diameter were counted. # means *P*<0.05 vs WtASCs (t-test, n = 5).

EAEASCs and WtASCs were assessed for their self-renewal ability by the colony forming unit (CFU) assay. All colonies greater than 2 mm in diameter were counted. The results indicate that EAEASCs (27.8±11.12 CFU) have the same self-renewal capacity as WtASCs (35.2±8.20 CFU) (Fig. 3A) (t-test, *P*>0.05). However, the colony diameter or area of WtASC was much larger and the intensity of the crystal violet stain was more intense when compared to EAEASCs (Fig. 3B). Elution of the stain with methanol, and determination of the optical density (OD) of the solution at 584 nm were performed to quantitate these differences. An arbitrary unit was defined as the OD divided by the pre-counted colony numbers. As shown in Figure 3C, WtASCs presented a much higher arbitrary CFU (4.51±0.73%) than EAEASCs (2.09±0.43%) (t-test, *P*<0.05).

### Cytokine and Chemokine expression of EAEASC

**Figure 4:**
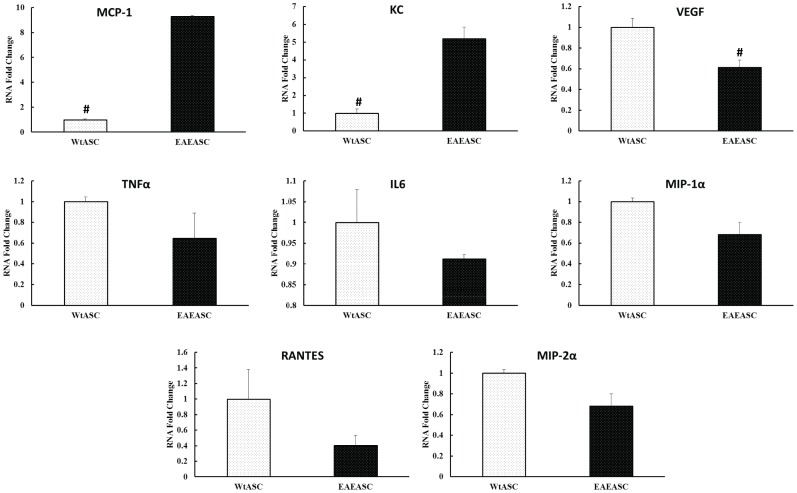
Real-time PCR analysis of cytokine and chemokine profiles of EAEASCs and WtASCs. Both cell types were cultured overnight, trypsinized, and analyzed by real-time PCR to determine the expression levels of various cytokines and chemokines TNFα, IL-6, MCP-1, MIP-1α, RANTES, KC, MIP-2α and VEGF. # means *P*<0.05 EAEASCs vs WtASCs (t-test, n = 3).

Both cell types were cultured overnight, trypsinized, and analyzed by real-time PCR to determine the mRNA expression levels of various cytokines and chemokines (TNFα, IL-6, MCP-1, MIP-1α, RANTES, KC, MIP-2α and VEGF). EAEASC demonstrated increased expression of MCP-1 and KC, and reduced expression of VEGF (t-test, *P*<0.05). There is no statistical difference between the two cell types related to their expression of TNFα, IL-6, MIP-1α, RANTES, and MIP-2α (Fig. 4) (t-test, *P*>0.05).

### Therapeutic effects of EAEASC on MOG_35–55_ induced chronic EAE

**Figure 5:**
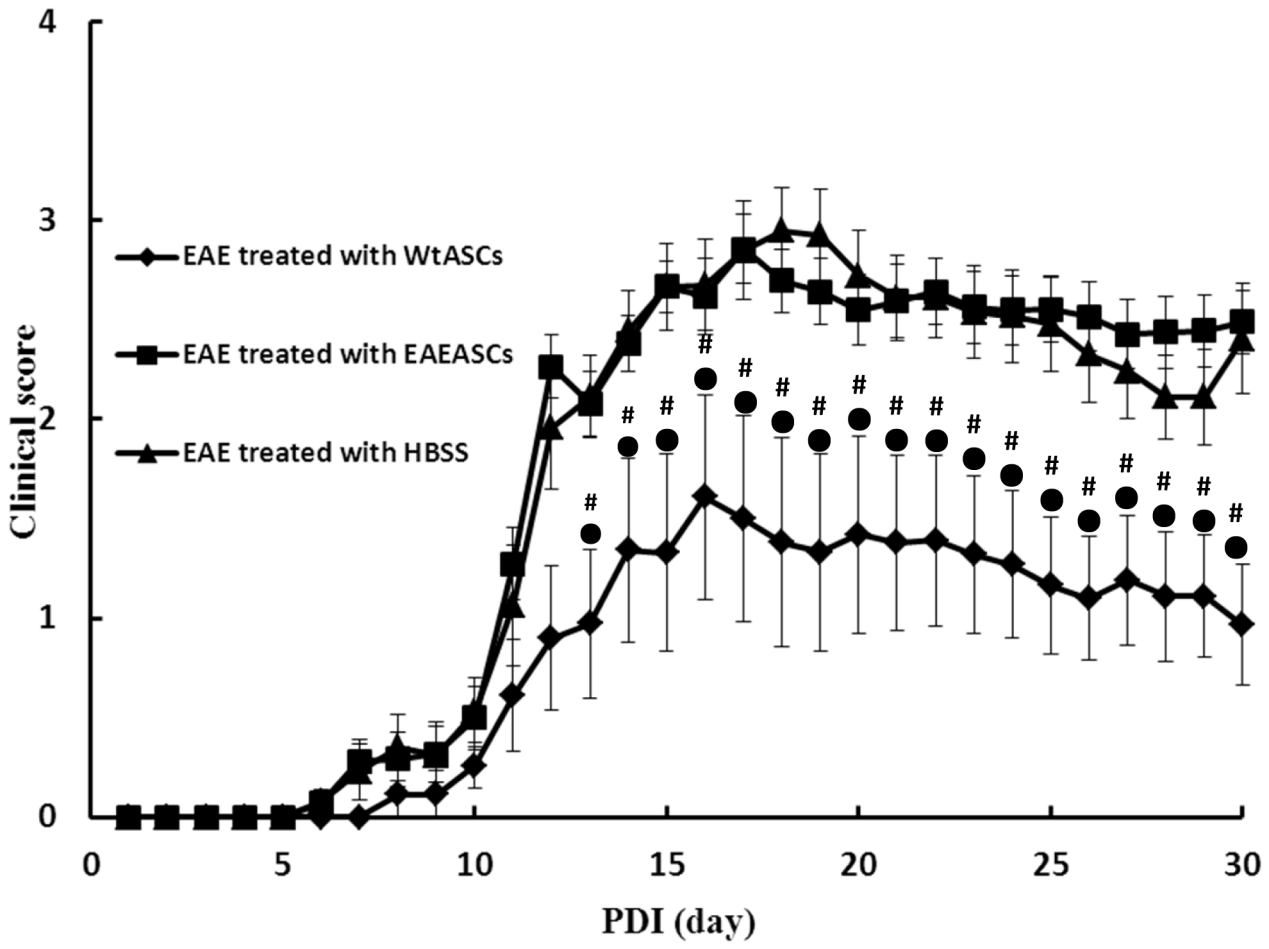
Clinical scoring for disease onset and progression. The clinical scores for the HBSS-treated (n = 20), EAEASC-treated (n = 20) and WtASC-treated (n = 20) EAE mouse groups were recorded daily for the duration of the study. WtASCs significantly reduced the clinical symptoms from PDI13 where the EAEASCs failed to mediate any therapeutic efficacy or improvement. # means *P*<0.01 vs HBSS-treated EAE group (post-hoc, n = 20); • means *P*<0.01 vs EAEASCs-treated EAE group (post-hoc, n = 20).

The therapeutic potential of EAEASCs was investigated in the EAE model. EAEASCs and WtASCs were administrated into the EAE mice by i.p. injection simultaneously on the day of disease induction. Our previous work (unpublished data) demonstrated that ASCs derived from healthy mice could significantly ameliorate the disease course and autoimmune mediated demyelination. Consistent with these prior studies, infusion of WtASCs ameliorated the symptoms and delayed the disease progression in comparison to the HBSS-treated controls, resulting in a statistically significant reduction of cumulative disease scores (Fig. 5). Moreover, mice treated with WtASCs showed a delayed onset of disease when compared with those in the HBSS-treated control group (Fig. 5). However, animals infused with EAEASCs showed no therapeutic improvement on the disease progression, and the mice in this group had an earlier onset of disease than those in the HBSS-treated control group.

### Histological and immunohistochemistry analysis on the spinal cord

**Figure 6:**
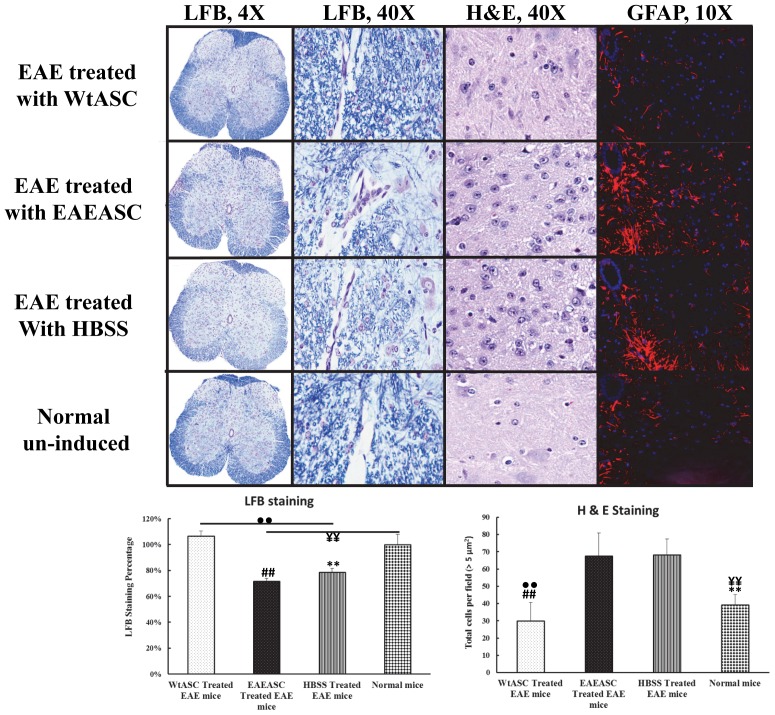
Histologic and Immunohistochemical analysis on the spinal cord. Spinal cords were collected at sacrifice from 3 mice per group. Each spinal cord was sectioned, mounted, and stained with luxol fast blue (LFB), H&E, and anti-GFAP for intact myelin, cell infiltration and astrocytes levels. Images were acquired at 4×, 40× and 10×magnification. The LFB stained sections (n = 3 mice; 3 sections/mouse) were quantified for the intensity of the blue staining using Aperio Software. The H&E stained sections (n = 3 mice; 3 sections/mouse) were quantified for the total cells per field using Fiji/Image J software. ## means *P*<0.01 EAEASCs-treated vs WtASCs-treated EAE group (post-hoc, n = 9); ** means *P*<0.01 EAEASCs-treated vs normal naïve mouse group (post-hoc, n = 9); ••means *P*<0.01 HBSS-treated vs WtASCs-treated EAE group (post-hoc, n = 9); **¥¥** means *P*<0.01 HBSS-treated vs normal naïve mouse group (post-hoc, n = 9).

**Figure 7:**
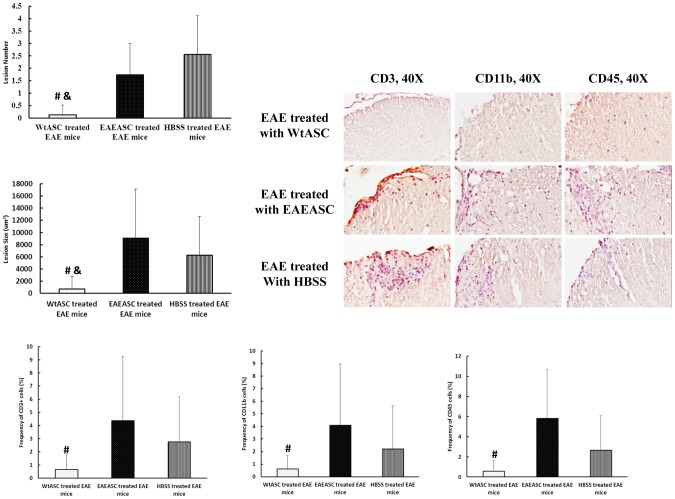
Lesion and cell infiltration analysis on the spinal cord. Spinal cords were collected at sacrifice from 3 mice per group. Each spinal cord was sectioned, mounted, and stained with anti-CD3, CD11b, and CD45 followed by Hematoxylin counterstain for lesion size, number and cell infiltration analysis. # means P<0.05 vs EAEASC-treated EAE group (post-hoc, n = 9); & means P<0.05 vs HBSS-treated EAE group (post-hoc, n = 9).

Mice from the treatment and control groups were sacrificed on PDI day 30, and the lumbar spinal cords were stained with LFB and H&E for the assessment of demyelination and cell infiltration respectively. Quantification of demyelination by density analysis of LFB-stained spinal cord sections revealed a 22% decrease in myelin density in HBSS-treated EAE mice. Furthermore, EAEASC treatment showed no improvement in re-myelination and had the same myelin density reduction degree (29%) as the HBSS-treated EAE mice (Fig. 6) (post-hoc, *P*>0.05). In contrast, WtASC treated animals had less demyelination, with myelin densities similar to those mice in the normal control (post-hoc, *P*>0.05). For cell infiltration analysis, as shown in Figure 6, EAEASC treatment did not reduce the levels of cell infiltration and had the same number of cells infiltrated (67.6±13.3 cells per field) as the HBSS-treated EAE mice (68.2±9.21cells per field), while WtASC treatment (30.0±10.6 cells per field) significantly decreased the number of infiltrating cells present in the spinal cords compared to the HBSS-treated and the EAEASC treated mice (post-hoc, P<0.01). Immunohistochemistry staining for the assessment of the cellular composition of the infiltrates revealed that there was increased astrocyte population in the EAEASC treated and the HBSS-treated mice as compared to the WtASC treated and the normal control mice (Fig. 6), Moreover, CD3, CD11b and CD45 staining demonstrated that there were more T cell, macrophage and B cell infiltration in the EAEASC treated mice as compared to the WtASC treated ones (Fig. 7). As shown in Figure 7, lesion analysis of the spinal cords also revealed that the lesion number and size were significantly reduced in the WtASC treated mice when compared to the EAEASC treated and the HBSS-treated ones.

### Serum cytokine levels

**Figure 8:**
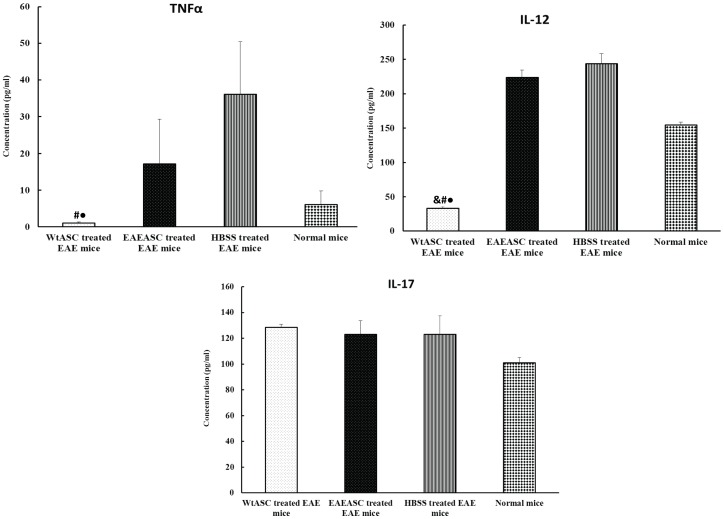
Pro-inflammatory cytokine protein levels in mouse serum. Sera from 5 mice per group at sacrifice were pooled and analyzed by ELISA to detect levels of TNF-α, IL-12, and IL-17. # means *P*<0.05 vs HBSS-treated EAE group (post-hoc, n = 5); • means *P*<0.05 vs EAEASCs-treated EAE group (post-hoc, n = 5); & means *P*<0.05 vs normal naïve mouse group (post-hoc, n = 5).

ELISA Assays were used to investigate the TNFα, IL-12 and IL-17 cytokines levels in each treatment group. As illustrated in Figure 8, WtASC treatment significantly reduced the circulating levels of pro-inflammatory cytokines TNFα and IL-12 (post-hoc, *P*<0.05) when compared to HBSS-treated EAE mice. Furthermore, the levels of TNFα and IL-12 were similar to (TNFα, post-hoc, *P*>0.05) or even lower (IL-12, post-hoc, *P*<0.05) than those circulating in the normal mice. However, the EAE mice treated with EAEASC had serum TNFα and IL-12 levels indistinguishable from HBSS-treated EAE mice (post-hoc, *P*>0.05). The circulating level of IL-17 remained the same in all the treatment and control groups (ANOVA, *P*>0.05).

## Discussion

Transplantation of BM-MSCs from healthy syngeneic controls has demonstrated striking therapeutic effects and unique immuno-modulatory capacities when delivered early to EAE mice [6], [27]. ASCs have similar properties to BM-MSCs, and have received increased attention in regenerative medicine and tissue engineering because of the abundance, easy accessibility of adipose tissues, and the feasibility of harvest cells by a minimally invasive procedure. Previous studies from our group [28] and other labs [14] have showed that intraperitoneal administration of ASCs derived from healthy mice could significantly ameliorate the disease course and autoimmune mediated demyelination through the regulation of the inflammatory responses. The data, showing milder disease symptoms with decreased number of inflammatory infiltrates and reduced demyelination, further confirmed the immune-modulatory effects of wild type ASCs. The results demonstrated that the therapeutic effect of WtASCs is due to the modulation of the inflammatory response associated with the tissue damage rather than tissue repair sustained by regeneration of damaged neurons and oligodendrocytes. The therapeutic benefits are related to immune suppression exerted by WtASCs as demonstrated in other studies [6], [27], [28].

In this paper, ASCs isolated from HBSS-treated EAE mice were cultured, analyzed *in vitro*, and intraperitoneally transplanted into EAE mice *in vivo* to investigate whether the disease altered the biology profiles of ASCs *in vitro* and the efficacy of autologous transplantation *in vivo*. The EAEASCs displayed all the characteristics that defined for MSCs *in vitro*. They are adherent to plastic surfaces, fibroblast-like and spindle shaped. The EAEASCs displayed the same characteristic surface markers. EAEASCs, like WtASCs, are positive for surface markers CD29 and ScaI, and negative for hematopoietic markers CD45, CD31, CD34 and macrophage marker CD11b. The EAEASCs could readily form single cell colonies, and differentiate into osteocytes and adipocytes when induced under proper media. However, the results showed that the osteogenic capacity of the EAEASCs was reduced as compared to the WtASCs (Fig. 2). The reason that EAEASCs have reduced osteogenic differentiation ability is unclear. There was no difference in osteogenic markers, such as alkaline phosphatase and runt-related transcription factor 2, in differentiated EAEASCs and WtASCs. Assessment of their inflammatory cytokine and chemokine profiles through real-time PCR demonstrated that the EAEASCs displayed a more pro-inflammatory profile with elevated expression of MCP-1 and KC as compared to the WtASCs. Studies have shown that chronic inflammation adversely affects bone formation [29], [30]. The pro-inflammatory profile of EAEASCs may play a role in their reduced differentiation capacity along the osteogenic lineage.

Interestingly, the EAEASCs demonstrated a lack of therapeutic efficacy when transplanted back into the EAE mice *in vivo*. The data showed that the EAEASCs did not delay the disease time course, decrease the extent of demyelination or decrease inflammation (Fig. 5–8). It is not clear why EAEASCs lacked a therapeutic benefit when delivered for the treatment of EAE mice. However, when tested for *in vitro* cytokine and chemokine expression levels, EAEASCs increased expression of MCP-1 in comparison to the WtASCs. MCP-1 (known as chemokine C-C motif ligand 2 in systematic nomenclature) is one of the key chemokines that regulate the migration and infiltration of monocytes/macrophages [31]. While constitutive expression of MCP-1 in healthy CNS is relatively low, it is present and plays a critical pathogenic role in mediating neuroinflammation in various diseases including MS and its EAE animal model [32]–[34]. The exact mechanism of MCP-1 action remains unclear, however, numerous studies [35]–[39] have recognized that MCP-1 modulates blood-brain barrier disruption and attraction of mononuclear leukocytes migration into the CNS. In rodents with EAE, MCP-1 expression is correlated with disease severity and parallels with disease onset [40], [41]. MCP-1 synthesis inhibition by the compound bindarit resulted in the delay, prevention and attenuation of EAE disease [34], which further supports the widely proposed MCP-1 role in neuroinflammation. It seems that, although they were cultured and expanded *in vitro* under standard cell culture condition, the EAEASCs may have been irreversibly altered by the diseased pro-inflammatory microenvironment *in vivo*, which may contribute to the lack of EAEASC therapeutic efficacy.

To date, clinical trials for MS have not focused on the use of autologous cells, but have used heterologous donor cells [42], [43]. Nevertheless, one study analyzed the characteristics of the BM-MSCs from MS patients in *vitro*, and it concluded that BM-MSCs from individuals with MS, compared to individuals without MS, displayed a normal phenotype and were similar in proliferation, *in vitro* differentiation potential and cell surface antigen expression [44]. Four Phase 1 clinical trials for the treatment of MS using autologous BM-MSCs have been approved [45]–[49]. The preliminary data from these trials demonstrated promising data in terms of clinical parameters and immunological analysis [46]. However, the early findings will require further validation with larger studies due to the limited number of the patients enrolled in the trials and the uncontrolled natures of the trials.

In conclusion, the ASCs from mice with EAE displayed a normal phenotype, typical MSC surface antigen expression, and *in vitro* osteogenic and adipogenic differentiation capacity; however, the degrees of osteogenesis and CFU formation were compromised relative to WtASC. Most importantly, the EAEASCs lost their therapeutic efficacy for the treatment of EAE mice *in vivo*, which may be due to the distinct pro-inflammatory secretory profile they developed in the setting of chronic neuroinflammation. Thus, autologous transplantation of ASCs for the treatment of the mouse model of EAE appears to lacking efficacy. While careful validation and experimental testing must be performed before any extrapolation is made based on the animal studies, the current work suggests that autologous ASCs from individuals with MS should be evaluated and compared extensively to allogeneic ASCs from healthy individuals prior to clinical trials.
